# The Correlation Between the Strength of the Shoulder and Trunk Muscular Systems in Elite Adolescent Water Polo Athletes

**DOI:** 10.7759/cureus.29775

**Published:** 2022-09-30

**Authors:** Spyridon Sioutis, Konstantinos Zygogiannis, Maria-Eleni Papakonstantinou, Ioannis Zafeiris, Fotini Soucacos, Pavlos Altsitzioglou, Apostolis Skouras, Dimitrios Karamintzas, Charilaos Tsolakis, Panagiotis Koulouvaris

**Affiliations:** 1 First Department of Orthopaedic Surgery, National and Kapodistrian University of Athens, Attikon University Hospital, Athens, GRC; 2 Department of Trauma and Orthopaedics, Laiko General Hospital, Athens, GRC; 3 Third Department of Paediatrics, National and Kapodistrian University of Athens, Athens, GRC

**Keywords:** youth sports, peak torque, trunk strength, shoulder strength, water polo

## Abstract

Introduction

Water polo is a competitive team sport played in the water between two teams of seven players each. Water polo players must have swimming speed, strong abdominal and back muscles, and strong shoulder muscles to cope with this sport's special conditions. In this study, we investigate the possible association of shoulder and trunk muscle systems in adolescent water polo athletes of high demands.

Materials and methods

The research included 42 water polo players aged 14-16, who train regularly for at least five years, six times a week, and participate in national championships and national teams. The athletes were evaluated on the strength and torque of these muscular systems using the isokinetic dynamometer Biodex System 4 Pro (Biodex Medical Systems, Inc, Shirley, NY). The correlation of the results was done using the statistical package SPSS 21.

Results

The correlations revealed statistically significant differences in trunk extension in combination with the shoulder external/internal rotation ratio. Also, most of the correlations occurred between the trunk and non-dominant limb of the athletes and, more often, in the female athletes. Furthermore, for the hand grip, the male athletes showed a greater difference in strength between the dominant and the non-dominant member than female athletes. Finally, the evaluation of the trunk extension/flexion ratio and external/internal rotation ratio for the shoulder joint showed that many athletes are outside the normal range and need targeted strengthening.

Conclusion

The negative correlation coefficient between trunk extension/flexion and shoulder external/internal rotation indicates that the trunk extension mechanism helps for better internal rotation of the shoulder. Therefore, water polo players should focus on the training of the stretching mechanism of the trunk and also give weight to achieving a balance between the competing muscular systems of the trunk and the shoulder. Thus, athletes can maximize their skills and, at the same time, protect themselves from injuries.

## Introduction

Water polo is a team sport that takes part in water with two teams of seven athletes each. Water polo was contained for the first time in the 1900 Olympic games in Paris and is now very popular in the USA, Canada, Australia, and Europe [1]. Water polo demands many physical skills and a good muscular system because the athletes must be excellent at swimming, exiting the water properly, shooting the ball accurately, and winning battles under the water [2]. Specifically, they must have a strong trunk (frontal abdominal wall and muscles of the back) in order to achieve under-body rotation. In addition, they should have a strong muscular system in the shoulder and the chest to effectively shoot the ball and be protected from overuse injuries. This is why they are characterized as overhead athletes [3,4]. Finally, they should also swim quickly so as to cover the needs of the game.

Studies for the shoulder kinematics for the dominant shoulder of water polo athletes show no significant differences in kinematics compared to usual people. It is also proven that shoulder internal and external rotation measurements can predict the possibility of a joint injury for athletes [5,6]. Additionally, the trunk is essential for water polo athletes because it enables them to rotate their bodies and exit the water alongside the lower limbs. In literature, there is a lack of studies examining the role of the trunk in water polo. Our aim for this study is to correlate the kinesiology of the trunk and shoulder in elite water polo athletes and describe the relationship.

In our study, we included elite adolescent water polo players with participation in the Hellenic national team. We performed specific measurements by the isokinetic dynamometer and evaluated the force, power, and torque of the shoulder and trunk muscular system. Specifically, we evaluate the internal and external rotation of the shoulder and extension and flexion of the trunk.

## Materials and methods

In our experimental design, we included 42 young athletes aged between 14 and 16 years old (mean age 14.8±0.8), who were examined with specialized measurements. Both sexes were selected for the study, 18 males and 24 females. These athletes were all members of the Hellenic Water Polo National Team U16 age levels. The athletes were usually training 5-6 times per week for the last two years before the measurements.

In the context of this study, we used the isokinetic dynamometer Biodex System 4 Pro (Biodex Medical Systems, Inc, Shirley, NY) [7], which is a modern multiform dynamometer with the ability to instantly record and analyze measurements on a built-in computer system and that can also be applied to all muscle group and joints. The trunk measurements were performed by measuring the peak torque per body weight and mean power of flexion and extension of the trunk at a stable angle of 90°. In addition, corresponding measurements were performed in the shoulder joint (dominant and non-dominant upper limb) at stable angles of 60° and 180°. These certain angles were chosen in order to simulate the kinesiology of normal in-water situations and to include the whole range of motion for the shoulder joint and the trunk.

The normal distribution of the measured values was examined using the Kolmogorov-Smirnov test. For the normally distributed variables, t-tests and ANOVA were used. The Mann-Whitney U test was used for variables that did not follow the normal distribution. The Pearson correlation coefficient (r) was used to study the correlations. Data were analyzed using SPSS statistical program version 23.0 (SPSS, Chicago, IL). All tests were double-sided. The p-value <0.05 was defined as the level of statistical significance. As the athletes were teenagers, their parents signed a consensus for their participation in our study and were approved by the scientific council of Attiko General University Hospital with ΕΒΔ 575/20-10-2020 number.

We also examined the anthropometric characteristics (age, height, weight, BMI, hand grip strength, upper limb length, and muscle mass) (Table 1).

**Table 1:** Anthropometric and physiological characteristics of the athletes who participated in the study.

Parameter	Mean	SD
Age	15.39	0.85
Height (cm)	172.50	9.08
Weight (kg)	70.23	13.70
BMI (kg/m^2^)	23.42	3.13
Upper limb length (cm)	177.93	10.94
Hand grip strength (kg)	40.60	12.84
Muscle mass (kg)	51.20	11.20
Heart rate (bpm)	69.40	13.90

## Results

The mean value of isometric peak torque at 90° for the trunk extension was significantly correlated (r= -0.312, p=0.044) with the corresponding mean value of the ratio of external/internal rotator muscles' peak torque of the non-dominant shoulder at 60°.

The mean value of isometric peak torque at 90° during trunk flexion was significantly correlated (r= -0.311, p=0.045) with the corresponding mean value of the ratio of external/internal rotator muscles' peak torque of the non-dominant shoulder at 60°.

The mean value of the ratio of the isometric peak torque at 90° along the trunk extension relative to the body weight of the athletes was significantly correlated (r=-0.382, p=0.012) with the corresponding mean value of the ratio of external/internal rotator muscles' peak torque of the non-dominant shoulder at 60°.

The mean value of the ratio of isometric peak torque at 90° during the flexion of the trunk in relation to the body weight of the athletes was significantly correlated (r=-0.324, p= 0.039) with the corresponding mean value of the ratio of external/internal rotator muscles' peak torque of the non-dominant shoulder at 60°.

No statistically significant correlations were observed for the dominant shoulder's external and internal rotator muscles at 60° with both trunk extension and flexion (range of motion 90°).

The mean value of isometric peak torque at 90° for the trunk extension was significantly correlated (r=-0.537, p<0.001) with both the mean value of the ratio of external/internal rotator muscles' peak torque of the dominant shoulder at 180° (r=-0.537, p<0.001) and the mean value of the corresponding ratio of external/internal rotator muscles' peak torque for the non-dominant shoulder at 180° (r=-0.707, p<0.001).

The mean value of isometric peak torque at 90° during the trunk flexion was significantly correlated (r=-0.378, p=0.015) with the corresponding mean value of the ratio of external/internal rotator muscles' peak torque of the dominant shoulder at 180°.

The mean value of the ratio of isometric peak torque at 90° along the trunk extension to the body weight of the athletes was significantly correlated (r=-0.382, p=0.014) with both the mean value of the external/internal rotator muscles' peak torque of the dominant shoulder at 180°, as well as the mean value of the corresponding ratio of the external/internal rotator muscles' peak torque in the non-dominant shoulder at 180° (r=-0.465, p=0.002).

No statistically significant correlation was observed for the relationship between external/internal rotator muscles in both the dominant and the non-dominant shoulder due to the isometric peak torque at 90° for the trunk flexion in relation to the body weight of the athletes.

Regarding the grouping of the specific measurements for the athletes according to their gender, in the examined male athletes, a statistically significant correlation (r=- 0,591, p = 0.022) was found between the mean value of the isometric peak torque at 90° in the extension of the trunk with the corresponding mean value of the ratio of the external/internal rotator muscles torque of the non-dominant shoulder at 180°. There was no other statistically significant difference in the extension/flexion of the trunk and the external/internal rotator muscles of the shoulder from the measurements made in male athletes.

The adolescent female water polo players who participated in this research study had more statistically significant correlations regarding the relationship between the trunk and the shoulder.

Specifically, the mean value of the isometric peak torque at 90° along the trunk extension was significantly correlated (r = -0.426, p = 0.043) with the corresponding mean value of the external/internal rotator muscles' peak torque of the non-dominant shoulder at 60°.

The mean value of isometric peak torque at 90° along the trunk extension was significantly correlated (r = -0.425, p = 0.038) with both the mean value of the external/internal rotator muscles' peak torque of the dominant shoulder at 180° and the mean value of the corresponding ratio of the external/internal rotator muscles peak torque for the non-dominant shoulder at 180° (r = -0.696, p<0.001).

The mean value of the ratio of isometric peak torque at 90° along the trunk extension to the body weight of the athletes was significantly correlated with both the mean value of the ratio of the external/internal rotator muscle peak torque in the non-dominant shoulder at 60° (r = -0.463, p = 0.023), as well as the mean value of the corresponding ratio of the external/internal rotator muscle peak torque in the non-dominant shoulder at 180° (r = -0.443, p = 0.030) (Table 2).

**Table 2:** The statistically significant correlations made with SPSS 21 concern the relationship between the mean value of the isometric peak torque for the trunk extension and flexion at a stable angle of 90° and the ratio of the mean value of the peak torque for the shoulder external/internal rotation at a stable angle 60° and 180° for both dominant and non-dominant upper limb of the athletes (r: Pearson correlation coefficient, p-value <0.05 was defined as the level of statistical significance).

	Trunk extension (90^o^)	P-value	Trunk flexion (90^o^)	P-value	Trunk extension per body weight (90^o^)	P-value	Trunk flexion per body weight (90^o^)	P-value
Ratio of shoulder external/internal rotation (60^o^): Non-dominant limb	r = -0.312	p = 0.044	r = -0.311	p = 0.045	r = -0.382	p = 0.012	r = -0.324	p = 0.039
Ratio of shoulder external/internal rotation (180^o^): Non-dominant limb	r = -0.707	p < 0.01			r = -0.465	p = 0.002		
Ratio of shoulder external/internal rotation (180^o^): Dominant limb	r = -0.537	p < 0.001	r = -0.378	p = 0.015	r = -0.382	p = 0.014		
			Male Athletes					
Ratio of shoulder external/internal rotation (60^o^): Non-dominant limb								
Ratio of shoulder external/internal rotation (180^o^): Non-dominant limb	r = -0.591	p = 0.022						
Ratio of shoulder external/internal rotation (180^o^): Dominant limb								
			Female Athletes					
Ratio of shoulder external/internal rotation (60^o^): Non-dominant limb	r = -0.426	p = 0.043			r = -0.463	p = 0.023		-
Ratio of shoulder external/internal rotation (180^o^): Non-dominant limb	r = -0.696	p < 0.001			r = -0.443	p = 0.030		
Ratio of shoulder external/internal rotation (180^o^): Dominant limb	r = -0.425	p = 0.038						

Asymmetries and differences between the sexes

Between male and female athletes, we identified a significant difference in the force of the dominant and non-dominant upper limbs. In particular, the young male water polo athletes show a significant difference, with the mean value of the dominant upper limb exceeding the force of the non-dominant upper limb by 5.50 N (52.89 N in the dominant upper extremity and 47.39 N in the non-dominant upper extremity). The female water polo athletes show a less significant difference, 1.08 N (31.33 N in the dominant upper extremity and 30.25 N in the non-dominant upper extremity). The manual hand-grip dynamometer was performed for the evaluation (Table 3).

**Table 3:** Mean value of the upper limb force (N). The manual hand-grip dynamometer was performed for the evaluation.

	Dominant	Non-dominant	Difference
Male	52.89	47.39	5.50
Female	31.33	30.25	1.08

Of the 42 water polo male and female athletes who participated in the study, 23 (54.76%) showed a ratio of trunk extensors/abdominal muscles' peak torque between 0.5 and 0.7, which is the normal range. Separately, by gender, 12 out of 18 (66.67%) female athletes and 11 out of 24 (45.83%) male athletes are out of the normal limits. Furthermore, nine athletes (21.43%) showed a result of <0.5, with the corresponding results by gender being two of the 18 male athletes (11.11%) and seven of the 24 female athletes (29.17%). A ratio bigger than 0.7 was presented in 10 of the 42 (23.81%) athletes who took part in the measurements, particularly four of the 18 males (22.22%) and six of the 24 females (25%). The mean values ​​of the ratio were 0.6128 for the boys, 0.6146 for the girls, and 0.6140 for the whole sample (Table 4).

**Table 4:** The percentage effects of the ratio of the mean value of the isometric peak torque for the trunk extension/flexion at a stable angle of 90°.

Ratio of mean value of isometric torque for the trunk extension/flexion (90^o^)	<0.5	0.5-0.7	>0.7
Overall	21.43%	54.76%	23.81%
Male	11.11%	66.67%	22.22%
Female	29.17%	45.83%	25%

The ratio of the external/internal rotator muscles' peak torque was evaluated in both the dominant and the non-dominant shoulder at a stable angle of 60°.

In the dominant shoulder, a ratio within the normal range was presented in 11 of the 42 (26.19%) members of the study, and specifically, two of the 18 boys (11.11%) and nine of the 24 girls (37.50%). Results smaller than the desired ratio of the external/internal rotator muscles were seen in 27 of the 42 (64.29%) athletes. Of the 18 athletes who took part in the measurements, 15 (83.33%) showed a ratio of <0.65. While of the 24 athletes, 12 (50%) had a lower than the desired ratio. Only four out of 42 (9.52%) athletes had shown a ratio of bigger than 0.75, one in 18 boys (5.56%) and three in 24 girls (12.5%). The mean values ​​of the ratio of the external/internal rotator muscles' peak torque for the dominant shoulder are 0.604 for the boys, 0.6486 for the girls, and 0.630 for the whole sample (Table 5).

**Table 5:** The percentage effects of the ratio of the mean value of the isometric peak torque for the shoulder external/internal rotation (dominant upper limb) at a stable angle of 60°.

Ratio of mean value of the isometric peak torque for the shoulder external/internal rotation (dominant upper limb) (60^o^)	<0.65	0.65-0.75	>0.75
Overall	64.29%	26.19%	9.52%
Male	83.33%	11.11%	5.56%
Female	50%	37.50%	12.50%

In the non-dominant shoulder, a ratio within the normal range was presented in 11 out of 42 (26.19%) athletes in the study, and in particular, five of the 18 males (27.78%) and six of the 18 females (33.33%). Results smaller than the desired ratio of the external/internal rotator muscles were identified in 26 of the 42 (61.90%) athletes. Of the 18 male athletes who took part in the measurements, 13 (72.22%) showed a ratio of <0.65 (72.22%). Also, of the 24 athletes, 13 (54.17%) had a smaller ratio. Of all water polo athletes, only five out of 42 (11.90%) showed a ratio of more than 0.75, and specifically, none of the male athletes (0.0%) and five out of 24 female athletes (20.83%). The mean values ​​of the ratio of the external/internal rotator muscles of the non-dominant shoulder are 0.5865 for male athletes, 0.6767 for female athletes, and 0.6380 for the whole sample (Table 6).

**Table 6:** The percentage effects of the ratio of the mean value of the isometric peak torque for the shoulder external/internal rotation (dominant upper limb) at a stable angle of 60°.

Ratιo of mean value of the isometric peak torque for the shoulder external/internal rotation (dominant upper limb) (60^o^)	<0.65	0.65-0.75	>0.75
Overall	61.90%	26.19%	11.90%
Male	72.22%	27.78%	0%
Female	54.17%	33.33%	20.83%

The strength and torque of the torso and shoulder were correlated. Statistically significant relationships emerged mainly in the torso extension in combination with the out/inward rotation ratio of the shoulder. The negative correlation coefficient (r) between the isometric force and the torque of the trunk at the angle at 90° with the mean value of the ratio of external/internal rotation of the shoulder for these specific parameters showed that the increased strength of the back contributes to better control and increased strength of the muscles which perform the internal rotation of the shoulder, a movement very important for water polo. More correlations in the relationship between the torso and shoulder strength were found in female athletes than in athletes and also in the non-dominant than in the dominant member. This fact is explained by the fact that boys have a more developed muscular system, thus being able to deepen in the targeted training of the member with which they shoot the ball in relation to the girls. Respectively, because the non-dominant member is given less importance in training, there is a tendency of the back to help the inner turn since the absence of specialized training allows the contribution of the back muscles of the torso to the inner turn of the shoulder to be seen better. Also, there was a significant difference in the strength of the dominant in relation to the non-dominant extremity during the hand-grip strength in the male athletes but not in the female athletes who took part in the study.

Measurements of the external/internal rotation ratio of the dominant shoulder in relation to the isometric strength showed that only 26.19% of the athletes were within normal limits. On the contrary, 64.29% showed a ratio lower than the minimum normal limit. This is due to the increased strength of the muscles that turn inward on the upper limb, as this is the movement to make the shot of the ball, which is very basic for water polo players. On the contrary, only 9.52% show asymmetry outside and inside the shoulder rotation, with the muscles that turn the arm outwards predominating. Therefore, these athletes will need to strengthen the inward-facing muscles of the shoulder joint to improve their performance on the ball. The results were similar in the non-dominant shoulder, with 26.19% of the athletes who participated in the research being within normal limits, 61.90% being below normal limits, and only 11.90% of water polo players being above normal limits.

## Discussion

In literature, great importance has been given to the recording and analysis of the anthropometric and physiological parameters of water polo players and to the function of the shoulder joint to achieve maximum efficiency and avoid overuse injuries [8,9]. Influenced by publications in high-level footballers, where a significant correlation emerged between the torso and the extensor mechanism of the knee, we chose to study whether there is a corresponding correlation in high-level water polo players in terms of the relationship between the world and shoulder movement [10]. Shoulder injuries are common for water polo players and the purpose of our study is the possible correlation of the trunk with the shoulder muscle groups so that through more intensive torso training and with exercises for the abdominal and back muscles, athletes gain greater stability and reduce asymmetries in the upper extremities.

From the statistical analysis of this sample of athletes, important conclusions emerge regarding the relationship between the trunk and the shoulder in water polo athletes. Initially, from the recording of the anthropometric characteristics of these athletes, it is shown that they are in good physical shape. In addition to the measurements made for the cardiorespiratory capacity of the athletes, it appeared that they have very good cardiovascular and respiratory system functions. Correlations were made in the measurements carried out for the strength and torque of the trunk and shoulder. Statistically significant differences emerged mainly in the extension of the trunk in combination with the ratio of external/internal rotation of the shoulder. The negative correlation coefficient (r) in these specific correlations between the isometric peak torque of the trunk at the extension at a 90° angle with the mean value of the external/internal shoulder rotation ratio for these parameters proves that the increased strength of the dorsal muscles that extend the trunk contributes to the strengthening and stability of the muscles that perform the internal shoulder rotation (subscapularis and pectoralis major). The internal rotation of the shoulder joint is very important for water polo players, as with this movement, they release the ball during the shot. The statistical analysis also showed that most correlations emerged between the trunk and the non-dominant member.

The majority of correlations between trunk and shoulder were recorded in female athletes. At the same time, there was a significant difference in the strength of the dominant upper limb in relation to the non-dominant limb during the hand-grip strength measurement in the male athletes but not in the female athletes who took part in the study. This asymmetry is caused by the targeted training and is observed in all sports where one of the two extremities is used to a much greater degree than the other. In top-level and demanding athletes, such as the participants in our study, it is important to evaluate the balance between competing muscles in addition to the strength of each muscle group [11]. This ensures the best possible prevention of injuries due to overuse and also gives the opportunity to coaches and trainers to create personalized training programs depending on the characteristics and weaknesses of each athlete. In our study, the ratio of the strength of the dorsal muscles of the trunk in relation to the abdominal muscles was evaluated, as well as the muscle group that makes an external rotation in the shoulder in relation to the muscles that perform an internal rotation, both in the dominant and non-dominant end. From the measurements made in the athletes, normal value ranges emerged for the ratio of extension/flexion of the trunk and for the ratio of external/internal rotation of the shoulder. The activity of the competing muscle groups was evaluated based on the normal margins. In collaboration with the water polo coaches, individualized training programs were developed so that the young athletes could find the ideal balance to improve their performance and minimize the risk of overdose. With such training, they can also work on their eccentric repetitive movement that occurs due to an imbalance of different muscular systems.

In the trunk, the abdominal muscles cause flexion, and the dorsal muscles extend the trunk. The normal ratio between these competing systems is defined as the interval between 0.5 and 0.7 (dorsal muscle strength/abdominal muscle strength) for measurements made on an isokinetic dynamometer at an angle of 60°. Corresponding measurements in swimmers and other overhead athletes have shown that the ideal trunk extension and flexion ratio is within similar limits [12]. In the shoulder, the different muscle groups turn the arm outwards and inwards, movements that are very important for handling the ball in water polo [11]. The normal ratio for the shoulder is defined as the interval between 0.65 to 0.75 (force of the external/internal rotators) in measurements that take place on the isokinetic dynamometer at an angle of 60°. In particular, in the shoulder, this asymmetry between competing muscle groups leads to eccentric movement, increasing the friction of the tendon of the supraspinatus with the acromion, leading to tendon tears from overuse injuries. The measurement of the ratio of the isometric force of the trunk for the extension and flexion in the examined athletes showed that 54.76% of them were within the normal limits, 21.43% were below the normal limits, and 23.81 % were above normal limits. About half of these athletes have good balance and stability in the trunk. Of the rest, half should focus on strengthening the dorsal muscles of the trunk, and the rest, those above normal limits, should follow a special training program for the abdominal muscles. The measurement of the external/internal rotation torque ratio of the dominant shoulder to the isometric force showed interesting results. Only 26.19% of the athletes were within normal limits, while on the contrary, 64.29% showed a ratio lower than the normal minimum limit. This is due to the increased strength of the muscles that turn inward on the arm, as this is the movement to make the shot of the ball, which is very basic for water polo players. In contrast, only 9.52% show asymmetry in external and internal shoulder rotation, athletes who have given the least basis to targeted shoulder training in water polo and who have increased muscle strength. The results were similar in the non-dominant shoulder, with 26.19% of the athletes who participated in the research being within normal limits, 61.90% being below normal limits, and only 11.90% of water polo players being above normal limits. From these results, it is evident that these young athletes lag significantly behind for the external rotation in relation to the internal rotation of the shoulder, something that their coaches should improve through individualized training programs in order to achieve optimal results and avoid possible injuries of the shoulder joint due to the eccentric movement. These results were presented and discussed with the head coaches of the national teams, enabling them to organize individual training programs for the trunk of these international athletes.

The hand-grip strength measurement of the athletes showed that the dominant upper extremity was stronger than the non-dominant, with a statistically significant difference. Furthermore, the difference was greater in boys than in girls due to their developed muscular system at the age of 14-16 years. This phenomenon is called exercise asymmetry and occurs in all sports, where athletes use one extremity more than the other (basketball, volleyball, water polo) [13,14].

Finally, we recorded the anthropometric characteristics (height, body weight, full development of the upper extremities, and BMI) and the cardiorespiratory capacity of the athletes who took part in this study. The results showed that these are quite developed children for their age and their measurements showed their excellent physical condition due to the daily intensive training. Anthropometric characteristics in water polo are a field of great importance and have been studied extensively in the literature [15-18]. Knowledge of this data helps coaches and athletes to know their advantages and weaknesses and to select which position they can perform best, how long they can withstand the great stress in the pool and what they need to improve to achieve better performance. In addition, water polo athletes have a great research interest, as these are athletes who, in order to cope with the action, will have to develop many different skills simultaneously (swimming, endurance, speed, trunk stability, strong shooting) [19].

Therefore, the coaches, having the statistical analysis and the advice of specialized health professionals of different specialties, can structure individualized training programs for each athlete. In this way, harmonious cooperation is created between the members of the scientific team, the athletes, and the coaches, which improves the daily life and the competitive performance of the young athletes [20]. Furthermore, when these measurements are repeated during the season, a more detailed profile of the athlete is built, and coaches are given the opportunity to know the status of their athletes at any time. This way, coaches can select the best-trained athletes to play a leading role and protect athletes with muscle imbalance from possible injuries.

The limitations of the study were the absence of measurements in the water element, which is the natural field where young water polo players compete, as well as the restriction of the movement of the humerus joint in the range of motion between 60° and 180° in the isokinetic dynamometer. Despite the limitations, the correlations of trunk and shoulder strength measurements helped draw useful observations about the performance, characteristics, and weaknesses of these elite adolescent water polo players. This specific research is recommended for this topic, as the literature is limited, and the knowledge of the trunk and shoulder correlations in water polo players has an important impact on water polo training programs [21].

## Conclusions

These data lead to the conclusion that more intense muscle development of adolescent athletes allows them to emphasize the strengthening of the dominant member in relation to their peer water polo athletes. This asymmetry is characterized by exercise-induced hypertrophy and is observed in all sports where one of the two extremities is used to a much greater degree than the other.

The statistical analysis of these parameters and the interdisciplinary cooperation between doctors, physiotherapists, and coaches are very important to identify each athlete's needs individually and build individualized training programs for each athlete. Furthermore, the repetition of the specific measurements during the season can give a complete profile of the athlete's condition and enables the coaches to make better use of the team members.
